# Smartphone Global Positioning System–Based System to Assess Mobility in Health Research: Development, Accuracy, and Usability Study

**DOI:** 10.2196/42258

**Published:** 2023-03-02

**Authors:** Robert P Spang, Christine Haeger, Sandra A Mümken, Max Brauer, Jan-Niklas Voigt-Antons, Paul Gellert

**Affiliations:** 1 Quality and Usability Lab Technische Universität Berlin Berlin Germany; 2 Institute of Medical Sociology and Rehabilitation Science - Charité Universitätsmedizin Berlin Berlin Germany; 3 Immersive Reality Lab University of Applied Sciences Hamm-Lippstadt Lippstadt Germany; 4 German Research Center for Artificial Intelligence (DFKI) Berlin Germany

**Keywords:** geographic information system, rehabilitation, prevention medicine, geoinformatics, out-of-home mobility

## Abstract

**Background:**

As global positioning system (GPS) measurement is getting more precise and affordable, health researchers can now objectively measure mobility using GPS sensors. Available systems, however, often lack data security and means of adaptation and often rely on a permanent internet connection.

**Objective:**

To overcome these issues, we aimed to develop and test an easy-to-use, easy-to-adapt, and offline working app using smartphone sensors (GPS and accelerometry) for the quantification of mobility parameters.

**Methods:**

An Android app, a server backend, and a specialized analysis pipeline have been developed (development substudy). Parameters of mobility by the study team members were extracted from the recorded GPS data using existing and newly developed algorithms. Test measurements were performed with participants to complete accuracy and reliability tests (accuracy substudy). Usability was examined by interviewing community-dwelling older adults after 1 week of device use, followed by an iterative app design process (usability substudy).

**Results:**

The study protocol and the software toolchain worked reliably and accurately, even under suboptimal conditions, such as narrow streets and rural areas. The developed algorithms had high accuracy (97.4% correctness, *F*_1_-score=0.975) in distinguishing dwelling periods from moving intervals. The accuracy of the stop/trip classification is fundamental to second-order analyses such as the time out of home, as they rely on a precise discrimination between the 2 classes. The usability of the app and the study protocol was piloted with older adults, which showed low barriers and easy implementation into daily routines.

**Conclusions:**

Based on accuracy analyses and users’ experience with the proposed system for GPS assessments, the developed algorithm showed great potential for app-based estimation of mobility in diverse health research contexts, including mobility patterns of community-dwelling older adults living in rural areas.

**International Registered Report Identifier (IRRID):**

RR2-10.1186/s12877-021-02739-0

## Introduction

From a functional perspective, mobility can be defined as the “ability to move oneself independently from one point to another” [1]. In the last years, broad conceptions of mobility that integrate individual mobility behavior (eg, mobility patterns) with environmental factors (eg, built environment or transportation modes) have gained importance [2,3]. Despite this development, in health sciences, most studies still assess mobility using self-report questionnaires that come along with self-reporting biases such as overestimating the time spent being active [2,4,5]. To allow for a more objective measurement of mobility, personal factors and environmental differences were integrated with data collected via a global positioning system (GPS) and data from geographic information systems (GISs) [6]. Especially, as the use of global navigation satellite systems such as GPS has become more reliable and less costly, the number of related studies has increased substantially. Among the various studies performed in this regard, researchers have examined the relationship between real-life mobility (eg, assessed through GPS) and health outcomes such as depressive symptoms [7,8], cognitive functioning [9,10], and general health status [11,12]. GPS/GIS-based mobility patterns further have the potential to inform about health behaviors such as physical activity outside the home and routines in mobility behavior (eg, time of the day first moved or revisited locations). Moreover, frameworks to guide the analysis under spatial and temporal aspects or attributes of movement (eg, active transport by foot or passive motorized transport) have been developed over time and include GPS-derived outcomes of life or activity space [13,14].

There are several devices with underlying server infrastructure and data analysis pipelines capable of GPS tracking, including GPS watches, smartphones, and trackers such as the frequently used Qstarz device (ie, BT-Q100XT; QStarz International Co, Ltd) [15].

However, several aspects, such as accuracy, data security, and offline use, must be considered when working with GPS devices. For instance, data by Lee et al [16] showed that although accuracy ranged widely across studies and devices, overall these devices can be considered good. To adequately assess environmental interaction and human mobility behavior (eg, attributes of location and revisited locations), precisely identifying visited locations and trips between these locations is crucial [17].

For data security, most devices use preexisting software, where server locations remain with the software provider, and scientists may not be able to adequately adapt or change the output [15]. In addition, not every GPS assessment device supports offline use, which may be required to offer solutions applicable in combination with high data-protection standards or assessment of GPS data in rural areas without an internet connection.

The usability of GPS sensors, including those implemented in mobile devices, has been shown to be high in diverse groups of participants, including schoolchildren [18], commuting working adults [19], or community-dwelling older adults. Nonetheless, technical and usability obstacles have been reported and must be evaluated in different areas and populations, including in health promotion, disease prevention, therapeutic, and rehabilitation settings and research.

The aim of this study is to demonstrate a multicomponent system for conducting GPS-based studies, including an easy-to-use, low-cost, and easy-to-adapt smartphone app, over longer sampling intervals without a permanent internet connection and respective analysis pipeline.

## Methods

### Overview

This study was conducted in the context of the MOBILE study (Mobility in Old Age by Integrating Health Care and Personal Network Resources in Older Adults Living in Rural Areas) funded by the German Federal Ministry of Education and Research (grant number 01GY1803), an interventional study focusing on promoting out-of-home mobility including GPS-based mobility outcomes. The development of the technical components in measuring GPS-based outcomes is described in this paper.

While developing the GPS for the study’s purpose, we describe 3 consecutive steps: (1) outline each component of the system (development substudy); (2) report an integration study evaluating the system’s capabilities to derive accurate variables about users’ activity behavior (accuracy substudy); and finally, (3) examine the user experience in a sample of community-dwelling older adults and describe an iterative app design process to further optimize the usability for this specific group of users (usability substudy).

### Development of the App and Analysis Pipeline

In the first step of developing the GPS we created the system architecture, which consists of an Android (Google Inc/Alphabet Inc) smartphone app, a remote server, and an analysis pipeline. All components are described in more detail in the following sections.

This architecture allows for much flexibility as it does not require particular hardware or privacy policies concerning server hosting. The server is hosted at Technical University Berlin, which ensures data security and GDPR (General Data Protection Regulation) conformity of the European Union.

### The GPS.Rec2.0 Mobile App

The mobile app (GPS.Rec2.0) can be deployed on most phones running Android versions 6.0 or above. We deliberately supported this rather dated operating system version, because it allows us to support a wide bandwidth of different devices. The app offers a simple interface to configure recording parameters such as sample frequency and GPS accuracy. It can be configured to automatically start in the background after a reboot, which is particularly useful for intervention scenarios in which participants need to charge the devices. Other than this, the users are not expected to interact with the app in any way. The app stores several millions of records on the internal memory of the device. As soon as an internet connection is established (eg, in the laboratory, after an intervention), it transfers all the records to a configured server destination. This way, researchers access the recorded data retrospectively and ensure privacy matters simultaneously (ie, no live tracking possible). This design also allows us to have minimum interaction so participants are not distracted in their everyday lives. Our study protocol foresees participants to plug the phone into a charger at home and take the phone with them whenever they leave the house. Other than that, no interaction with the phone is necessary. In addition, this setup allows us to use comparably inexpensive smartphone hardware. We performed our tests on ZTE’s Blade A5 (2019), a basic, entry-level smartphone costing around €50 (US $54). When deciding on a hardware platform, we tested several devices offering at least 16-GB memory space, 1-GB RAM, and 1000 mAh battery capacity. The latter is probably the most critical specification as it allows the system to run and continuously record data even when participants forget to charge it for 1 night. Further, reducing the GPS query frequency helps to reduce battery consumption. We set a 10-second interval for acquiring position data.

In addition to recording GPS position data, the app records physical motion using the 3-axis accelerometer. The physical motion data are added to the analysis pipeline and further improve data quality as they help to distinguish between motion and stillness. The sampling frequency was set to 1 Hz, which was found to be suitable for our purpose. The smartphone app is free software under the GNU General Public License version 3.0.

### Server Backend

The backend server is a dockerized Ruby on Rails app offering 2 main components. First, it acts as a backend for the smartphone and provides a REST API (representational state transfer application programming interface) to retrieve recorded data from the study smartphones once they are back in our laboratories. This communication is SSL (secure sockets layer) encrypted using Let’s Encrypt certificates (Internet Security Research Group). As we provide the software in a dockerized format, researchers can deploy this backend quickly on their servers and need not rely on any third-party service. This way, we ensure compliance with local privacy policies.

**Figure 1:**
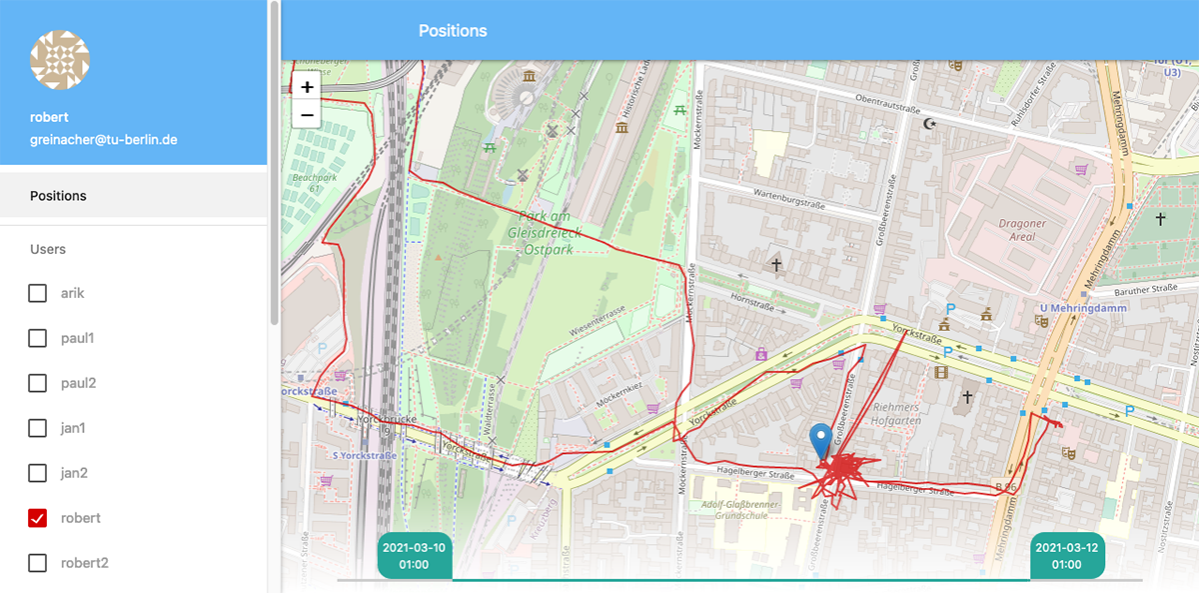
Screenshot of the server components user interface. It provides a straightforward assessment of the raw records given by a user ID and a time interval.

The second component of the server app is a user interface for visualizing the raw records obtained from different users participating in the study (Figure 1). Here, users and time intervals can be filtered, visualized, and directly downloaded as a CSV (comma-separated values) file for further processing and analysis. This tool is particularly useful for visual feedback if data are received and if the selected time interval contains the expected information. Although this interface is potentially reachable from the internet, users need to authenticate themselves using the same credentials (username and password) needed to log-in to the mobile app. The server backend is free software under the GNU General Public License version 3.0.

### Analysis Pipeline

The data analysis is provided as Python3 (The Python Software Foundation) libraries. Although fully featured GIS tools such as ArcGIS Pro (Esri) or QGIS (QGIS Development Team) exist, we decided to create a new analysis pipeline for faster batch processing up to several hundred study data sets. This streamlines the process and provides better accuracy, as we will demonstrate in the “Results” section.

The analysis is based on the Stop & Go Classifier, which identifies stop and trip intervals within the data set. As most mobility variables are based on this first distinction rather than raw GPS point clouds, this is the fundamental first step. For example, variables such as the number of “revisited places,” “time out of home,” or the “time spent in transit” can be directly constructed after an initial stop/trip interval detection. However, other metrics, such as the “perimeter of the convex hull,” are constructed based on individual GPS points instead of only a list of identified important locations.

**Figure 2:**
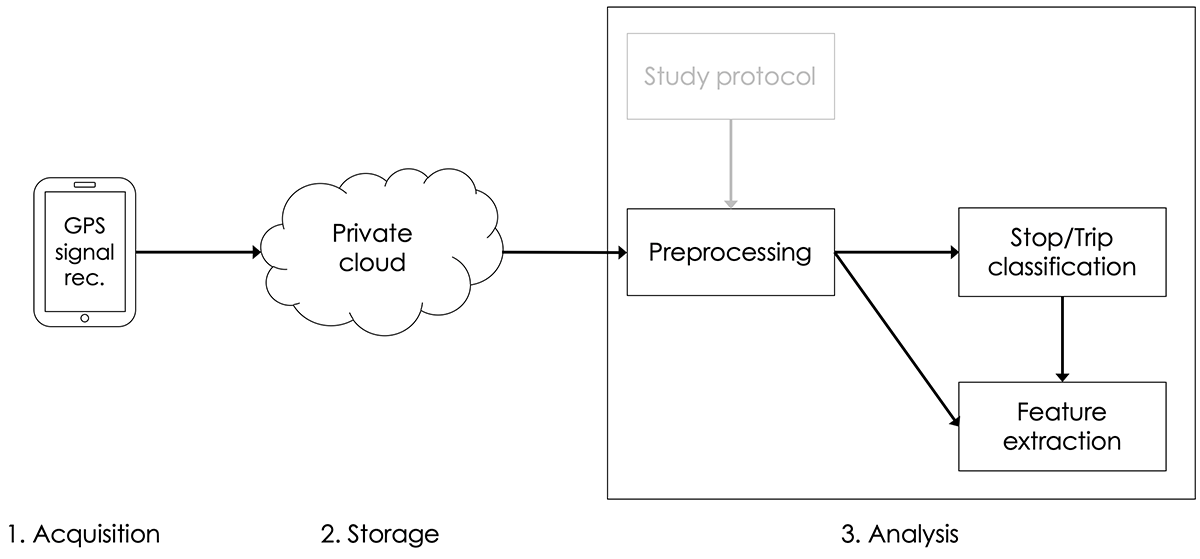
Flowchart of the data acquisition, storage, and processing steps. The analysis phase consists of several subtasks to determine the correct study interval, preprocess the raw GPS (and accelerometer) records, run the stop/trip classification, and combine its results into a feature vector of the variables of interest. GPS: global positioning system.

Figure 2 visualizes the data flow through the analysis pipeline. First, the data are recorded on a mobile device and stored locally. Later, when securely connected to the internet, the mobile app copies the recorded data to our private cloud, the server app. The server securely stores all samples (GPS and accelerometer data) from all users over the entire study period. If necessary, this provides a central access point for analyses, even for multiple analysts. The third phase is data analysis. We first consult the study protocol to carve out the study period for each user precisely. This is a necessary technicality because we shipped the configured study smartphones to the participants via postal service for this study. As a result, the supplied phones often also recorded the shipping routes. Therefore, our participants were instructed to start using the phone 1 day after receiving it through the postal mail. Thus, for the analysis, it was necessary to trim the start and end dates of the recorded data according to the actual study dates. Using the correct study dates, we accessed the web server and downloaded only records in the interval of interest. The downloaded data contained raw GPS and accelerometer samples and several status information of the recording device (eg, battery status and time stamps). In the preprocessing phase, basic filters are applied, such as removing duplicates and converting the accelerometer records into a motion score that describes the physical motion the recording device underwent at any given moment [20]. Lastly, the data are fed into the Stop & Go Classifier described earlier to identify trips and stops before the final set of features is extracted from all data available.

The analysis pipeline is designed in such a way that it reads a given CSV containing GPS and (optionally) accelerometry data, processes these, and outputs several result tables. These contain a list of all the important locations in a data set (ie, the stop intervals) and a detailed analysis of all variables of interest per day.

Table 1 provides a list of potential variables that may interest health researchers using the presented mobility analysis software framework. The list distinguishes variables based on all GPS samples and integrated variables built up using the stop/trip detection metrics.

**Table 1:** A list of all measures observed and calculated from the raw GPS^a^ and acceleration data collected during the trial.

Source and variable		Description
**Based on all samples**		
	Trip	Period of movement
	Stop	>5 minutes at the same place (within a radius of 100 m)
	Maximum distance from home (daily)	Maximum radius from home per day
	Average distance from home (daily)	Average distance from home per day
	Area standard ellipse (daily)	The minimum span ellipse that can fit all of the positions of the data set that is computed using a minimum covariance estimator [9,13,21]
	Area convex hull (including perimeter, surface, compactness)	Life-space measure [13,22]; see Figure 3
	Daily revisited life space %	Percentage of the daily convex hull that has overlap with any convex hulls of the other included study days
	Average revisited life space %	Average percentage overlap of the daily convex hull with the convex hulls of the other included study days
	Daily path area	Daily path area (DPA) is created by buffering each individual’s GPS trip with a 200-m buffer zone, then dissolving all buffered trips into 1 polygon and removing bodies of water [16,23]
**Based on stop/trip intervals**		
	Home	Home address, special case of stop
	Number of locations (daily)	Stop counts per day
	Number of revisited locations (daily)	Stop counts per day
	Number of unique locations (daily)	Unique stop counts per day
	Daily duration (daily)	Time out of home
	Time on foot/bike	Total trip time done by foot/bike [13,24]
	Time in vehicle	Total trip time done by car/public transport [13,24]
	Average time at home (daily)	Average time spent at home [9]
	Time of the day first move	Time of the day of first trip
	Time most moved	Time of the day of most trips in the categories morning/noon/evening
	Revisited paths %	Percentage of identical trips among all trips
	Entropy in location	Entropy is a measure for time distribution over different stop locations. A higher entropy either indicates a more regular time distribution with a higher number of locations or a higher number of locations [7].

^a^GPS: global positioning system.

**Figure 3:**
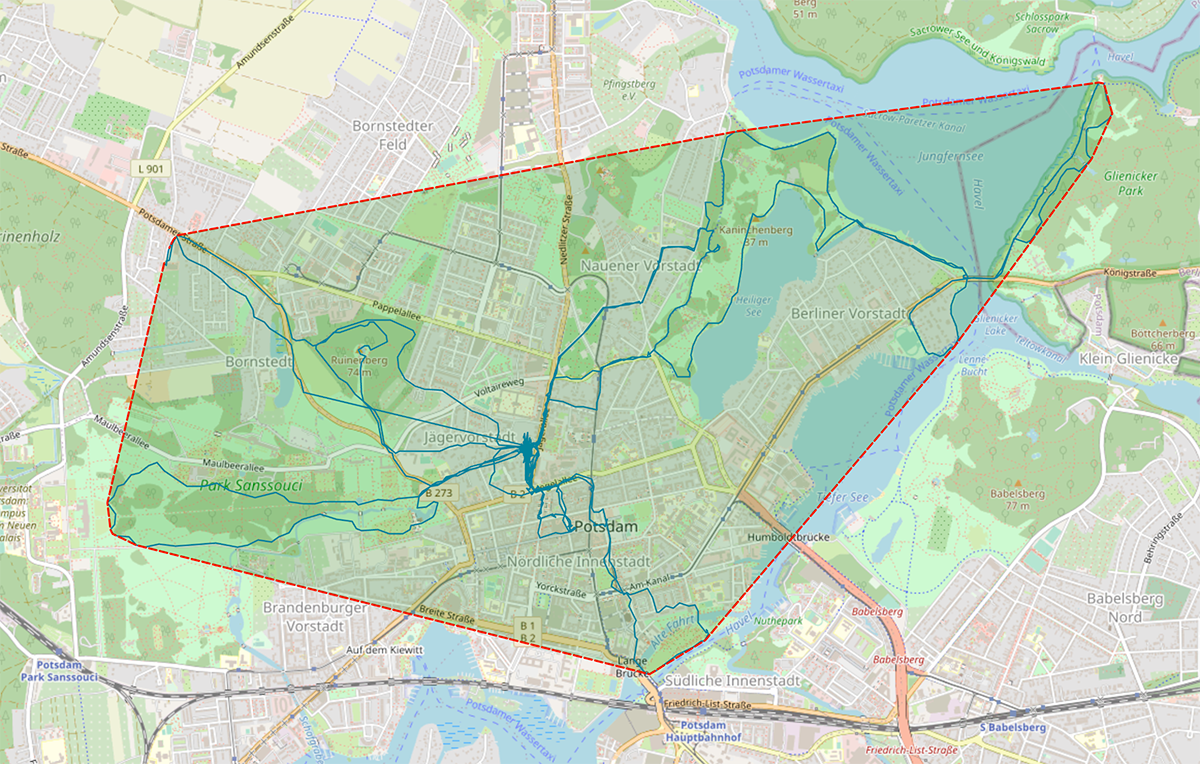
Hull curve around the position records of the whole example time span.

### Accuracy Evaluation Using a GPS Diary

To evaluate the validity of our analysis scripts, we conducted a field test dedicated to collecting GPS and acceleration data under realistic conditions and combined these data with a diary study. While the devices ran as we used them in the field (parameters described in the “The *GPS.Rec2.0* Mobile App” section), the diary contains changes of place, reference positions, and the beginning and ending of each stay. This way, we can compare the recorded data and their analysis with the ground truth of the testers involved. To obtain the most accurate reference data, we created a diary app (iOS app, run on iPhone XR) to log whenever a test person enters or leaves a position. This way, we can ensure precise tracking and digitally obtain time stamps and position data. For accuracy analysis, we focused on comparing exact timings of location changes, position deviations, durations of movement, and durations of dwell.

The diary contained 3 pieces of information per record: the beginning and end time of a stop, coordinates of the location (ie, longitude and latitude), and a reverse lookup address for easier identification of the samples. Position and time stamp are the only information we need to validate the automatic stop/trip detection of the GPS records.

Having stop intervals in the diary as ground truth, we labeled each GPS record from the mobile app as either “stop” or “trip.” Simultaneously, we ran the analysis pipeline to classify the raw, unlabeled GPS data set. This allowed us to obtain 2 sets of labels based on the diary and algorithmic analyses, which we can use to quantify the goodness of the classification.

### Usability in a Sample of Community-Dwelling Older Adults

As the *GPS.Rec2.0* app is being used in the MOBILE study with older adults (age ≥75), handling of the smartphone and app was tested in a usability study with a convenience sample of 9 participants (6 women and 3 men that were between 71 and 83 years of age and lived in a rural area of Brandenburg, Germany). The sample size was oriented on similar studies such as that by Brusilovskiy et al [25], who used GPS technologies for community health engagement, or Price et al [26], who performed a validation study for different GPS devices. The usability study was conducted between July and September 2020 after the first wave of COVID-19 infections in Germany. During this time, restrictions on social contact were still high as no vaccination was available yet, and therefore all components of the usability study were accomplished without personal contact. Participants received the smartphone with an installed preversion of the *GPS.Rec2.0* app, study information, consent paper, and a postservice usability questionnaire (Multimedia Appendix 1).

In addition, they were contacted via telephone and informed about how to turn on the phone, ensure that the app was running, and sufficiently charge the battery by charging the smartphone overnight, as well as requested to take the smartphone with them on every trip outside for 7 consecutive days. For additional usability, smartphones were prepared with stickers indicating the needed functions (eg, where to charge the smartphone or how to turn it on). After the testing phase, participants sent back the phone, consent paper, and questionnaires. Further, they were interviewed about their experiences within a structured phone call. Data have been analyzed descriptively as well as with content analysis. The content analysis categorizes interview content to examine patterns in communication in a noninvasive manner [27].

### Ethical Approval

Ethical approval was obtained by the Charité Ethics Commission embedded in the broader MOBILE study with the case number EA1_052_20 on May 14, 2020. In the ethics statement the implementation of a pilot study, including GPS device testing, interviews, and questionnaires, was explicitly listed.

## Results

### Development of the App and Analysis Pipeline

All components were developed iteratively and tested regularly. Apart from feature tests, testing the integration between smartphones, backend, and analysis was most important during the development process. Over the entire development period, we implemented regular field tests to identify design or implementation issues early on. The final app can record large numbers of position samples over a long period, even without a stable connection to the synchronize destination/backend. The backend can synchronize multiple clients simultaneously and cope with intermittent uploads (eg, disrupted internet connection during the upload process). As a fallback strategy, the mobile app can export GPX (GPS Exchange Format) files of the recorded data. This covers severe problems with the syncing process without losing any data.

In addition, the mobile phone’s battery life lasted at least two days and presented itself as suitable for the study.

We developed the analysis pipeline simultaneously, allowing us to iterate fast and reproduce design decisions of the backend on the analysis pipeline (eg, functions to fetch data via APIs). Furthermore, this allowed us to test outcomes and quantify recording and analysis accuracies as soon as possible in the development process.

### Accuracy Evaluation Using a GPS Diary

Over 4 months between October 2021 and May 2022, we recorded 692 stops using the GPS diary app (5.5 stops/day). During the same period, the *GPS.Rec2.0* app recorded 122,808 GPS samples (969.7/day; 1 every 89.1 seconds). This data set is publicly available. To compare the diary records with the results of our analysis pipeline, we used 2 approaches to quantify the system’s accuracy. Based on true positives (sample programmatically identified as a stop, which is a stop according to the diary), true negatives (sample identified as a trip and was recorded on a trip), false positives (identified as a stop but was a trip), and false negative (identified as a trip but was a stop), we analyzed balanced accuracy values (0.965) and *F*_1_-scores (0.975). Besides that, the system can correctly label 97.40% (119,614/122,808) of all samples.

While examining at the sample level is important to compare classification performance with other classifiers, it seems suitable to further examine analysis performance based on actual stop intervals—as these are the measure of interest at this stage. Furthermore, this allows the evaluation of systematic errors more easily than simple sample-by-sample comparisons. Hence, we aggregated the algorithmically obtained labels per sample to form intervals of stops and trips. Out of the 692 stops known to the diary, the system detected 667 stops; 97.3% (649/667) of these detected stops were identified correctly (corresponding to a similar time interval within the diary). The system, however, failed to identify 26 stops and 33 trips. Compared with the ground truth diary recordings, 19 diary stops were fragmented: a fragmented stop is detected as a set of several individual stops instead of 1 stop capturing the entire duration. This is an important metric, as many subsequent mobility assessment analyses buildup on these raw detected stops (eg, the average number of significant locations per day per person is computed using the total number of stops). Table 2 lists all relevant classification results of our accuracy substudy.

**Table 2:** Classification performance of our Stop & Go algorithm that was used to distinguish dwelling intervals (stops) from transit intervals (trips).^a^

Performance	Stop & Go classification including motion score	Stop & Go classification without motion score
Correct, n/N (%)	119,614/122,808 (97.40)	118,865/122,808 (96.79)
Balanced accuracy	0.965	0.966
*F*_1_-score	0.975	0.966
Stop counts (system/dairy), n	667/692	708/692
Missed stops, n	26	26
Fragmented stops, n	19	43
Trip counts (system/dairy), n	667/691	708/691
Missed trips, n	33	28
Runtime (seconds)	33.12	49.31

^a^Our algorithm can include accelerometer data to further refine results (ie, “motion score”); however, most conventional stop/trip classifiers do not offer such a feature. For better comparability with other systems, we reported results for both with and without accelerometer data.

### Usability in a Sample of Community-Dwelling Older Adults

Results of the questionnaire are reported in Table 3 and indicate that the smartphone was easy to integrate into the everyday life of the older adults interviewed. Participants reported little worries about data security or damage to the cell phone (8/9, 89%, fully agreed), followed by worries about battery level, damage, takeaway, and comprehensibility (6/9, 67%, fully agreed in all cases). In the qualitative interviews, participants described the need for a small belt bag to always carry the smartphone around, especially during summer activities such as gardening, shopping, or riding a bicycle.

**Table 3:** Questionnaire results of the usability substudy (n=9).

Variables	Disagree, n (%)	Rather disagree, n (%)	Somewhat agree, n (%)	Fully agree, n (%)
Joy: I enjoyed the 7 days of testing.	N/A^a^	N/A	5 (56)	4 (44)
Integration: The use of the GPS^b^ device is easy to integrate into my everyday life.	N/A	1 (11)	3 (33)	5 (56)
Time and activity: While using the GPS device, I need more time for my daily activities outside the home.	4 (44)	1 (11)	3 (33)	1 (11)
Battery level: The battery level lasts long enough for everyday use.	N/A	1 (11)	2 (22)	6 (67)
Damage: I am afraid of damaging the GPS device.	6 (67)	2 (22)	1 (11)	N/A
Privacy: I think that my personal data collected with the GPS device are properly protected.	N/A	1 (11)	2 (22)	6 (67)
Takeaway: I always remember to take the GPS device with me when I leave the house.	N/A	1 (11)	2 (22)	6 (67)
Comprehensibility: The external labeling of the GPS device is easy to understand.	N/A	N/A	3 (33)	6 (67)
Charging: The GPS device is easy to charge.	N/A	1 (11)	N/A	8 (89)
Help: When problems occur with the GPS device, I know whom to contact for problem solving.	N/A	1 (11)	N/A	8 (89)
Usefulness: The data collected by the GPS device are useful for (health) science.	N/A	N/A	4 (44)	5 (56)
Time: Filling out the questionnaires took too much time.	5 (56)	1 (11)	2 (22)	1 (11)

^a^N/A: not applicable (ie, no participant responded in the category).

^b^GPS: global positioning system.

## Discussion

### Principal Findings

This study aimed to develop a smartphone-GPS–based system for mobility analyses in health research and to test this system for accuracy and usability. Based on the experience of expert and user stakeholders with the proposed system for assessing GPS, it shows great potential for app-based estimation of mobility in community-dwelling older people.

The main findings of this study are that the developed GPS-based system works well for mobility analyses as the app functions without technical difficulties and performed well even under suboptimal conditions. Furthermore, the algorithm achieves high accuracy and its usability was piloted with older adults, which demonstrated low barriers and easy implementation. The system includes the following: the *GPS.Rec2.0* app, a backend for centralized data storage, and an associated analysis pipeline for the automatized transformation of raw GPS data into predefined variables. The app showed good accuracy in the accuracy substudy with staff members and good usability in successive tests in the usability substudy, which involved a sample of community-dwelling older adults living in a rural area.

The comparison between the system with and without the accelerometer data in Table 2 shows the most dominant advantage in the fragmented stops metric. As the recording device’s physical motion helps reduce the number of fragmented stops by more than half (43 vs 19), the number of identified stops is crucial for many mobility and daily activity indicators. Hence, reducing fragmented stops is an important objective, as fragmented stops artificially inflate the number of stops. Our system, the signal processing Stop & Go algorithm, helps to interpret GPS data more accurately. This component can be used independently from our other components, as it is released as a stand-alone open-source library [20].

### Comparison With Prior Work

In terms of accuracy, our study showed good-to-very good stop/trip identification results, which are comparable with other studies investigating the accuracy in other systems (see Spang et al [28] for a comparison of algorithms). One key element of any mobility analysis system is the algorithm for classifying trips and stops, which is the foundation for further mobility analyses. In terms of performance, our system showed higher accuracy, *F*_1_-score, more true stops and trips, and fewer false stops and trips than similar systems (MovingPandas and scikit-mobility). Although our system outperformed the reference systems in most areas, the number of missed trips was detected better using MovingPandas. Concerning the usability of the system, most participants indicated that the system is easy to implement in everyday life, which is in line with findings from other GPS usability studies in diverse urban adults [19] showing high levels of GPS acceptability and usability as well as low levels of wear-related concerns. Likewise, a study on patients with cardiac issues from urban and rural areas found low barriers and high ease of use; however, especially in rural areas, the periodic signal interruption was reported [29]. Thus, our approach provides a feasible tool not only in urban but also in rural areas and for older adults who are often excluded from GPS studies.

### Strengths and Limitations

This study has several strengths, including the mixed methods stepwise approach across substudies and the innovative system pipeline. However, some limitations need to be considered when interpreting these findings. First, although we developed an open-source system ready to use, performing a new study would require considerable resources and technical know-how. We tried to mitigate this by providing detailed descriptions about the source codes’ repository websites. This should make it easier to adapt the tools we developed to new research projects and swiftly test ideas. Second, we presented high levels of accuracy. Nonetheless, misclassifications occurred, and thus, in the future further algorithms are necessary to improve the classification performance even further. We are actively developing the classification module of the described system as a separate open-source contribution. As such, we are working on parameter tuning tools to provide easy and flexible setups, even for sensors or sampling rates different from what we used. This should further improve the reliability of the described toolchain. Third, we tested the usability in community-dwelling older adults; thus, although the system is quite generic and can likely be applied to a variety of settings and populations, we cannot rule out any usability issues in other populations. Future studies should test the system’s usability in other health contexts and cohorts, including urban areas or outpatient rehabilitation setting and the labor force or students.

Two main advantages lie within our system. First, the app was constructed for offline use, which has several positive attributes (ie, longer battery life, high data protection, no live-tracking possible), and thus has benefits over commercial GPS apps that include mobile data. The second advantage is that the open source development of the app includes the hosting of data on university servers rather than relying on existing commercial systems (eg, Qstarz or Garmin Forerunner; compare [15] with potential limitations to data protection). It ensures maximum data and privacy security and lets scientists alter the system architecture if necessary.

Although this proposed system was developed for the use case in health research with older adults, we assume that our systems also work well in different study populations such as schoolchildren or people with impairments. Furthermore, we believe our system is suitable for various study designs, such as observational or interventional studies.

### Code Availability

The *GPS.Rec2.0* app [30] is available as free software under a GNU General Public License version 3.0. The backend component [31] for storing, visualizing, and accessing recorded position and accelerometer samples of the *GPS.Rec2.0* app is also available.

The classification component of the analysis pipeline is available as an independent component, the Stop & Go Classifier. It is free software under a BSD 3-Clause license.

The test data set [32], used to evaluate the classification data set, was recorded using the described *GPS.Rec2.0* app. The data set contains GPS and acceleration records as well as stop/trip annotations. It is publicly available at the Open Science Framework under a CC-By Attribution 4.0 International license.

### Future Directions and Conclusions

In future GPS-based health studies, the system and its algorithms should be evaluated in a clinical study and analyzed with respect to clinical, subjective, and behavioral measures. We explicitly see potential for use in interventional studies, as it is a great tool to evaluate interventions that, for instance, focus on promoting out-of-home mobility, fostering new routines, changing mobility habits, or following patients after cardiac rehabilitation. As an individualized/tailored approach is used often (compare [33,34]), we believe it is possible to develop our system even further and add live feedback options for the user. Overall, GPS-based measurements can add great value to various study designs and populations and should be considered and examined more often in health research.

## Appendix

Multimedia Appendix 1 Questionnaire assessing usability of the GPS device. GPS: global positioning system.

## Abbreviations

CSV: comma-separated values
DPA: daily path area
GDPR: General Data Protection Regulation
GIS: geographic information system
GPS: global positioning system
GPX: GPS Exchange Format
MOBILE: Mobility in old age by integrating health care and personal network resources in older adults living in rural areas (research project name)
REST API: representational state transfer application programming interface
SSL: secure sockets layer
