# Supplementary material for: Smartphone Global Positioning System–Based System to Assess Mobility in Health Research: Development, Accuracy, and Usability Study
Source: JMIR Rehabil Assist Technol. 2023 Mar 2;10:e42258. doi: 10.2196/42258 (PMC10020906; doi:10.2196/42258)
Supplement: Multimedia Appendix 1 [file rehab_v10i1e42258_app1.docx]

**Questionnaire C – Opinion about GPS device**

Mobile in Havelland

**Please fill out once after the 7-day GPS-measurement**


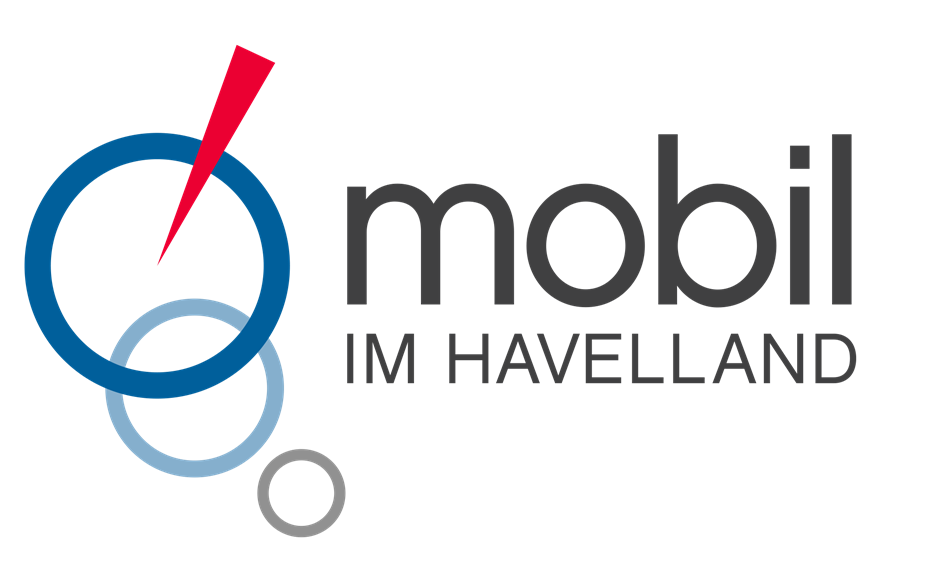


Thank you for testing our GPS-device. You’re almost there!

We kindly ask you to fill out a concluding questionnaire about using the GPS-device. Please choose one answer for each question.

**While answering the questions, please keep the 7-days of the GPS-testing in mind.**

| **I enjoy the 7-days of testing.**  *(please choose one answer)* | | | |
| --- | --- | --- | --- |
| ❑ | ❑ | ❑ | ❑ |
| Disagree | Rather disagree | Somewhat agree | Fully agree |

| **The use of the GPS device is easy to integrate into my everyday life.**  *(please choose one answer)* | | | |
| --- | --- | --- | --- |
| ❑ | ❑ | ❑ | ❑ |
| Disagree | Rather disagree | Somewhat agree | Fully agree |

| **While using the GPS device, I need more time for my daily activities outside the home.**  *(pleas choose one answer)* | | | |
| --- | --- | --- | --- |
| ❑ | ❑ | ❑ | ❑ |
| Disagree | Rather disagree | Somewhat agree | Fully agree |

| **The battery lasts long enough for everyday use.**  *(please choose one answer)* | | | |
| --- | --- | --- | --- |
| ❑ | ❑ | ❑ | ❑ |
| Disagree | Rather disagree | Somewhat agree | Fully agree |

| **I am afraid to damage the GPS device.**  *(please choose one answer)* | | | |
| --- | --- | --- | --- |
| ❑ | ❑ | ❑ | ❑ |
| Disagree | Rather disagree | Somewhat agree | Fully agree |

| **I think that my personal data, collected with the GPS device, I properly protected.** *(please choose one answer)* | | | |
| --- | --- | --- | --- |
| ❑ | ❑ | ❑ | ❑ |
| Disagree | Rather disagree | Somewhat agree | Fully agree |

| **I always remember the take the GPS device with me when I leave the house.**  *(please choose one answer)* | | | |
| --- | --- | --- | --- |
| ❑ | ❑ | ❑ | ❑ |
| Disagree | Rather disagree | Somewhat agree | Fully agree |

| **The external labelling of the GPS device is easy to understand.**  *(please choose one answer)* | | | |
| --- | --- | --- | --- |
| ❑ | ❑ | ❑ | ❑ |
| Disagree | Rather disagree | Somewhat agree | Fully agree |

| **The device is easy to charge.**  *(please choose one answer)* | | | |
| --- | --- | --- | --- |
| ❑ | ❑ | ❑ | ❑ |
| Disagree | Rather disagree | Somewhat agree | Fully agree |

| **When problems occur with the GPS device, I know whom to contact for problem solving.**  *(please choose one answer)* | | | |
| --- | --- | --- | --- |
| ❑ | ❑ | ❑ | ❑ |
| Disagree | Rather disagree | Somewhat agree | Fully agree |

| **The data collected by the GPS device is useful for (health) science.**  *(please choose one answer)* | | | |
| --- | --- | --- | --- |
| ❑ | ❑ | ❑ | ❑ |
| Disagree | Rather disagree | Somewhat agree | Fully agree |

| **Filling out the questionnaires took too much time.**  *(please choose one answer)* | | | |
| --- | --- | --- | --- |
| ❑ | ❑ | ❑ | ❑ |
| Disagree | Rather disagree | Somewhat agree | Fully agree |

**If there is more you want to tell us, feel free to do so.**

| **Is there something we can improve?** |
| --- |
| .......................................................................................................................................................................................................................................................................................................................................................................................................................................................................................................................................................................................................................................................................................................................................................... |

| **Anything else you want to tell us.** |
| --- |
| .......................................................................................................................................................................................................................................................................................................................................................................................................................................................................................................................................................................................................................................................................................................................................................... |


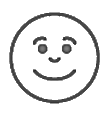
**Thank you!**
